# Chimeric antigen receptor containing ICOS signaling domain mediates specific and efficient antitumor effect of T cells against EGFRvIII expressing glioma

**DOI:** 10.1186/1756-8722-6-33

**Published:** 2013-05-09

**Authors:** Chan-Juan Shen, Yu-Xiu Yang, Ethan Q Han, Na Cao, Yun-Fei Wang, Yi Wang, Ying-Ying Zhao, Li-Ming Zhao, Jian Cui, Puja Gupta, Albert J Wong, Shuang-Yin Han

**Affiliations:** 1 Translational Research Center, Zhengzhou University People’s Hospital, #7 Weiwu Road, Zhengzhou, Henan 450003, China; 2 Drexel University College of Medicine, Philadelphia, PA 19129, USA; 3 Department of Neurosurgery, Brain Tumor Research Laboratories, Stanford University Medical Center, Stanford, CA 94305, USA

**Keywords:** Adoptive immunotherapy, Chimeric antigen receptor, Glioma

## Abstract

**Background:**

Adoptive transfer of chimeric antigen receptor (CAR)-modified T cells appears to be a promising immunotherapeutic strategy. CAR combines the specificity of antibody and cytotoxicity of cytotoxic T lymphocytes, enhancing T cells’ ability to specifically target antigens and to effectively kill cancer cells. Recent efforts have been made to integrate the costimulatory signals in the CAR to improve the antitumor efficacy. Epidermal growth factor receptor variant III (EGFRvIII) is an attractive therapeutic target as it frequently expresses in glioma and many other types of cancers. Our current study aimed to investigate the specific and efficient antitumor effect of T cells modified with CAR containing inducible costimulator (ICOS) signaling domain.

**Methods:**

A second generation of EGFRvIII/CAR was generated and it contained the EGFRvIII single chain variable fragment, ICOS signaling domain and CD3ζ chain. Lentiviral EGFRvIII/CAR was prepared and human CD3^+^ T cells were infected by lentivirus encoding EGFRvIII/CAR. The expression of EGFRvIII/CAR on CD3^+^ T cells was confirmed by flow cytometry and Western blot. The functions of EGFRvIII/CAR^+^ T cells were evaluated using in vitro and in vivo methods including cytotoxicity assay, cytokine release assay and xenograft tumor mouse model.

**Results:**

Chimeric EGFRvIIIscFv-ICOS-CD3ζ (EGFRvIII/CAR) was constructed and lentiviral EGFRvIII/CAR were made to titer of 10^6^ TU/ml. The transduction efficiency of lentiviral EGFRvIII/CAR on T cells reached around 70% and expression of EGFRvIII/CAR protein was verified by immunoblotting as a band of about 57 kDa. Four hour ^51^Cr release assays demonstrated specific and efficient cytotoxicity of EGFRvIII/CAR^+^ T cells against EGFRvIII expressing U87 cells. A robust increase in the IFN-γ secretion was detected in the co-culture supernatant of the EGFRvIII/CAR^+^ T cells and the EGFRvIII expressing U87 cells. Intravenous and intratumor injection of EGFRvIII/CAR^+^ T cells inhibited the in vivo growth of the EGFRvIII expressing glioma cells.

**Conclusions:**

Our study demonstrates that the EGFRvIII/CAR-modified T cells can destroy glioma cells efficiently in an EGFRvIII specific manner and release IFN-γ in an antigen dependent manner. The specific recognition and effective killing activity of the EGFRvIII-directed T cells with ICOS signaling domain lays a foundation for us to employ such approach in future cancer treatment.

## Background

Adoptive immunotherapy (AIT) has received much attention as a form of cancer treatment in the past decades. The adoptive transfer of the ex vivo cultured tumor-infiltrating lymphocytes or lymphokine-activated killer cells has been shown to mediate tumor regression in certain type of cancers [1]. However, AIT has only been used as adjuvant therapy in clinical practice, instead of the first-line treatment, due to some limitations. With advances in genetic engineering, immunotherapy using genetically modified antigen specific T cells, has become more attractive in the treatment of human malignancies. Chimeric antigen receptor (CAR), consisting of a single chain variable fragment (scFv) of a tumor antigen specific antibody and the signaling domains of T cell receptor (TCR), is one of the strategies to genetically modify T cells [2]. This strategy combines the antigen specificity of antibodies and the cytotoxicity of cytotoxic T lymphocytes (CTLs). CAR bypasses many of the mechanisms through which tumor cells escape immunorecognition, e.g., down-regulation of MHC and costimulatory molecules, induction of suppressive cytokine and regulatory T cells, etc. Recent studies have generated some encouraging preclinical and clinical data regarding the CAR-mediated adoptive immunotherapy in a variety of cancers including chronic lymphocytic leukemia, neuroblastoma and melanoma [3-5].

According to two-signal model of T cell activation [6], complete T cell activation and the prevention of activation induced cell death require costimulatory signal like CD28-B7 in addition to TCR/CD3 complex. The new generation of CAR with the addition of costimulatory molecules greatly strengthens its antitumor effects in vitro and in vivo. Many reports showed that the enhanced antitumor activity is due to the activation, proliferation and survival of the CAR T cells containing costimulatory molecules [7]. To date, the most commonly used costimulatory molecule in CAR is CD28, one of the best-characterized costimulatory molecules. CD28 costimulation is essential for IL-2 production, proliferation and survival of T cells, but thought to be less important in memory and effector T cell responses [8]. Inducible costimulator (ICOS), a B7 receptor family member similar to CD28 in structure, is expressed on activated T cells. Some studies have demonstrated high cytotoxicity and more favorable Th1/Th2 cytokine production due to costimulation by ICOS [9].

Both optimal design of the CAR architecture and careful choice of the tumor associated antigen are important prerequisite for attaining significant response in the CAR-mediated immunotherapy. Epidermal growth factor receptor variant III (EGFRvIII) is an oncogenic variant frequently expressed in glioma and many other types of cancers [10]. EGFRvIII is made of an in-frame deletion of exons 2–7 of EGFR, resulting in a truncated extracellular ligand binding domain and a constitutively activated protein in a ligand-independent manner. The expression of EGFRvIII is associated with survival, invasion, angiogenesis and resistance to radiation and chemotherapy in cancers, making it an attractive target for cancer immunotherapy [11]. Currently, targeting of EGFRvIII using strategies such as immunotoxin, vaccination, and/or small molecular inhibitor are actively carried out in both preclinical studies and clinical trials in many laboratories [12,13].

In this study, a second generation CAR, namely, EGFRvIII scFv-ICOS-CD3 ζ (hereafter named as EGFRvIII/CAR), was constructed based on our previously generated EGFRvIII specific recombinant antibody [14]. Lentivirus–mediated T cell transduction was used to modify human T cells. The genetically engineered T cells demonstrate a specific and efficient cytotoxicity against EGFRvIII expressing glioma in vitro and in vivo.

## Methods

### Construction of chimeric antigen receptor and generation of EGFRvIII expressing U87cell line

**Figure 1:**
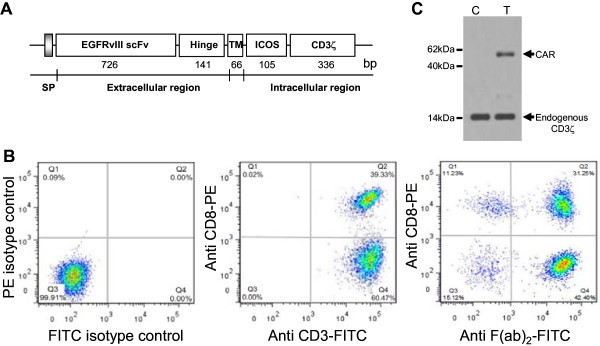
**Evaluation of EGFRvIII/CAR expression on CD3**^**+ **^**T cells.** (**A**) Schematic representation of EGFRvIII/CAR. It consists of EGFRvIII scFv, the hinge and transmenbrane (TM) region of human CD8α, ICOS signaling domain, and human CD3ζ chain. IgG κ chain was used as signal peptide (SP). (**B**) Surface expression of EGFRvIII/CAR on CD3^+^ T cells. Left, isotype control; Middle, anti-human CD3-FITC and anti-human CD8-PE staining (BD Biosciences); Right, anti-mouse F(ab)_2_-FITC (eBiocience) and anti-human CD8-PE staining. (**C**) Immunoblot analysis of EGFRvIII/CAR expression. Lysates of untransduced T cells (lanes 1) and EGFRvIII/CAR transduced T cells (lane 2) were separated by SDS-PAGE under reducing condition. Goat anti-human CD3ζ antibody was used to detect the endogenous and chimeric CD3ζ expression.

Chimeric EGFRvIII/CAR is composed of EGFRvIII scFv and ICOS-CD3ζ expression cassette. The EGFRvIII scFv was derived from a high-affinity EGFRvIII monoclonal antibody described previously with the order of a light chain -(GGGS)_3_- a heavy chain (726 bp) [14]. ICOS-CD3ζ expression cassette consisting a hinge and a transmembrane (TM) region was designed and synthesized by Anji Biotechnology Company, as shown in Figure 1A. The two fragments of EGFRvIII scFv and ICOS-CD3ζ were connected in-frame by overlap PCR. The generated EGFRvIII/CAR was verified by DNA sequencing and cloned into EcoRI and BamHI sites of lentiviral vector pCDH-EF1-T2A-puro (System Biosciences, CA). The new vector was named pCDH-EGFRvIII/CAR. The sequences of all PCR primers are provided on request.

Human glioma cell line U87 was bought from Chinese Academy of Science in Shanghai and maintained in Dulbecco’s modified Eagle’s medium (DMEM, Invitrogen, CA) supplemented with 10% FBS, 50 U/ml penicillin and 50 μg/ml streptomycin. The EGFRvIII expressing U87 cell line was generated by selecting stably transfected pcDNA3.1-EGFRvIII of U87 cells and cultured in DMEM containing 10% FBS and 400 μg ⁄ml G418.

### Preparation of lentiviral EGFRvIII/CAR

Lentiviral EGFRvIII/CAR was generated by calcium phosphate cotransfections of HEK293T cells with three plasmids (pVSV-G, pCMV-dR8.9 and pCDH-EGFRvIII/CAR) as described previously [15]. Harvested supernatants of transfected cells containing lentiviral particles were filtered and concentrated by ultracentrifugation. The titers were determined by QuickTiter™ Lentivirus Quantitation Kit (Cell BioLabs).

### Transduction of activated CD3^+^ T cells with lentiviral EGFRvIII/CAR

The CD3^+^ T cells were activated and transduced with lentiviral EGFRvIII/CAR as described previously [16]. Briefly, peripheral blood mononuclear cells (PBMCs) were isolated by Lymphoprep (Solarbio) gradient centrifugation. After washing and equilibrating with MACS buffer, T cells were isolated using CD3^+^ magnetic beads (Miltenyi Biotec). CD3^+^ T cells at [5×10^5^ cells/ml] were then activated with CD3/CD28 Dynabeads (Life Technologies) for three days. The activated CD3^+^ T cells were harvested and hereafter expanded in the presence of IL-2 (30 units/ml) for 7 days. Then, the activated CD3^+^ T cells were infected with lentiviral EGFRvIII/CAR at MOI of 5 in the presence of IL-2 (30 units/ml). After 96h, cells were collected for further analysis. All blood samples were obtained from healthy volunteers under an institutional review board-approved protocol.

### Evaluation of EGFRvIII/CAR expression on CD3^+^ T cells

Flow cytometric analysis was used to detect surface expression of EGFRvIII/CAR on CD3^+^ T cells. Cells were washed once with PBS containing 2% FBS/0.1% sodium azide and then incubated with the corresponding fluorescent antibody (2 μl/3×10^5^ cells) for 30 min at 4°C in the dark. After that, the cells were washed again and fixed in 0.5% paraformaldehyde/FACS buffer before analysis. Flow cytometric analyses were performed on BD FACSAria II with CellQuest Pro software. In all cases, >10,000 events was analyzed with antibody isotype control.

Western blot was done to verify the expression of EGFRvIII/CAR protein. T cells (3×10^6^) were lysed in 100 μl lysis buffer. After centrifugation, cell lysates were denatured under reducing condition and electrophoresed by 12% SDS-PAGE. The sample was then transferred to PVDF membrane (Millipore) and immunoblotted with goat anti-human CD3 ζ antibody (Santa Cruz Biotechnology). The blot was incubated with horseradish peroxidase-conjugated rabbit anti-goat IgG (Sigma) and detected by ECL Western Blotting Analysis System (Alpha Innotech).

### Functional analysis of EGFRvIII/CAR^+^ T cells

Cytotoxicity assay was done as described [15]. Briefly, 1×10^6^ target cells were labeled with 0.1 mCi (3.7 MBq) ^51^Cr and mixed with decreasing numbers of effector cells at effector-to-target ratios (E:T) of 40:1, 20:1, 10:1 and 5:1. After 4 h incubation, supernatants were collected and radioactivity was measured in a WIZARD2 gamma counter (Perkin-Elmer). The mean percentage of specific lysis of triplicate wells was calculated according to the following formula: (test release - spontaneous release)/(maximal release - spontaneous release) × 100. GFP^+^ T cells and non-transduced (NT) T cells were used as control of effector cells.

Cytokine release assay was performed by a co-culture of 2×10^5^ T cells with 1×10^6^ target cells in 200 μl medium per well in 96-well-plate triplicately. After 24 h, supernatants were assayed for IFN-γ production using ELISA (R&D Systems). The control of effector cells is the same as above cytotoxicity assay; the control of target cells is EGFRvIII-negative parental U87 cells.

### In vivo antitumor activity of EGFRvIII/CAR^+^ T cells

Xenograft tumor mouse model was established by subcutaneous (s.c.) flank injections of 5×10^6^ EGFRvIII expressing U87 cells in 6-week-old female BALB/cA-nude mice (Chinese Academy of Science Shanghai Experimental Animal Center). When the tumor burden reached about 500 mm^3^ in about 10–14 days after tumor cells inoculation, the mice were assigned to different groups (5 in each group) and injected with 1×10^7^ different T cells/100 μl (EGFRvIII/CAR-transduced T cells, GFP-transduced T cells, and control PBS) either either systemically to tail vein or locally to the tumor mass. Tumor growth was subsequently monitored by caliper measurement and tumor volume was calculated using the formula: 1/2 *× length ×* (*width*)^2^. The mice were killed when tumor volume reached >2,000 mm^3^. This study was carried out in strict accordance with the recommendations in the Guide for the Care and Use of Laboratory Animals. The protocols were approved by the Animal Care Committee of Zhengzhou University (Protocol No. 011–026).

### Statistical analysis

ANOVA was used to identify the possible difference among different treatment groups. Once the difference is confirmed, Student’s *t* test was applied to calculate the significance in the difference between two treatment groups (*P* values). *P*-Values less than 0.05 were considered statistically significant.

## Results

### EGFRvIII/CAR was constructed and T cells were modified successfully by lentiviral EGFRvIII/CAR

To generate EGFRvIII-specific T cells, chimeric EGFRvIII/CAR was constructed As shown in Figure 1A, EGFRvIII/CAR encodes a fusion protein consist of IgG κ leader peptide, EGFRvIII scFv, the hinge and TM region of human CD8α (amino acids 135–205), intracellular signal domain of ICOS (amino acids 165–199) and the CD3ζ chain (amino acids 52–163). No extra linker or space was used between gene fragments since it may increase the immunogenicity of EGFRvIII/CAR leading to immune destruction of the transduced T cells in vivo.

Lentiviral EGFRvIII/CAR was prepared for transduction of CD3^+^ T cells. The titers of Lentiviral EGFRvIII/CAR ranged from 1×10^6^ to 10×10^6^ transducing units/ml determined by QuickTiter™ Lentivirus Quantitation Kit (Cell BioLabs). After CD3^+^ beads selection of human PBMCs, the purity of CD3^+^ T cells were almost 100%, with 39.33% CD8^+^ T cells and 60.47% CD4^+^ T cells as indicated in Figure 1B (middle). The surface expression of EGFRvIII/CAR on T cells was confirmed by flow cytometric analysis using anti-mouse F(ab)_2_-FITC. As shown in Figure 1B (right), EGFRvIII/CAR expression efficiency reached 73.65% of CD3^+^ T cells, of which 31.25% were CD8^+^ T cells (CTLs). The transduction efficiency was usually about 70%. The whole population of EGFRvIII/CAR transduced T cells was treated as EGFRvIII/CAR^+^ T cells for subsequent experiments.

The EGFRvIII/CAR protein expression was verified by immunoblotting. Cell lysates of EGFRvIII/CAR transduced and untransduced T cells were separated by SDS-PAGE under reducing condition and immunoblotted with goat anti-human CD3ζ antibody. As shown in Figure 1C, under reducing conditions, endogenous CD3ζ chain was detected as a 15 kDa band in both transduced and untransduced T cell lysates. Additional band of approximate 57 kDa were observed in EGFRvIII/CAR transduced T cells but absent in untransduced T cells, consistent with the calculated size of EGFRvIII/CAR protein.

### EGFRvIII/CAR^+^ T cells demonstrated specific and efficient cytotoxicity against EGFRvIII expressing glioma cells

**Figure 2:**
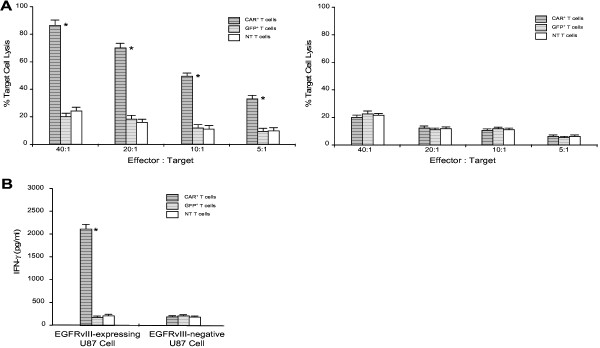
**Functional analysis of EGFRvIII/CAR**^**+ **^**T cells.** (**A**) Cytotoxic activity of EGFRvIII/CAR^+^ T cells. (Left) Target cells were EGFRvIII-expressing U87 cells which can be lysed by EGFRvIII/CAR^+^ T cells, not by GFP^+^ and NT T cells. (Right) Target cells were EGFRvIII-negative U87 cells, which can not be lysed by either EGFRvIII/CAR^+^ or control T cells. (**B**) Cytokine release of EGFRvIII/CAR^+^ T cells. Only EGFRvIII/CAR^+^ T cells released significant amount of IFN-γ when co-cultured with EGFRvIII-expressing U87 cells. No increased IFN-γ expression was detected when co-cultured with EGFRvIII-negative U87 cells, nor from control GFP^+^ and NT T cells. Results are the mean and the SD from experiments in triplicate. * indicate *P*<0.05.

A standard 4-hour ^51^Cr release assay was performed to determine whether the EGFRvIII/CAR^+^ T cells could recognize and kill the EGFRvIII expressing U87 cells. A robust enhancement in the cytotoxicity of the EGFRvIII/CAR^+^ T cells against the EGFRvIII expressing glioma cells was detected as an increase in E:T ratio, A significant difference was noticed in the EGFRvIII specific killing at each E:T ratio (P<0.05, Figure 2A, Left) between EGFRvIII/CAR^+^ T cells and control GFP^+^ or non-transduced T cells. The killing activity exceeded 60% at 10:1 of E:T ratio. In contrast, no evident killing activity was found among T cells of three different groups toward control target cells (EGFRvIII-negative U87 cells, Figure 2A, Right). This result confirmed the specificity and efficiency of cytotoxic T cell response against EGFRvIII expressing glioma cells when EGFRvIII/CAR was grafted onto T cells.

**Figure 3:**
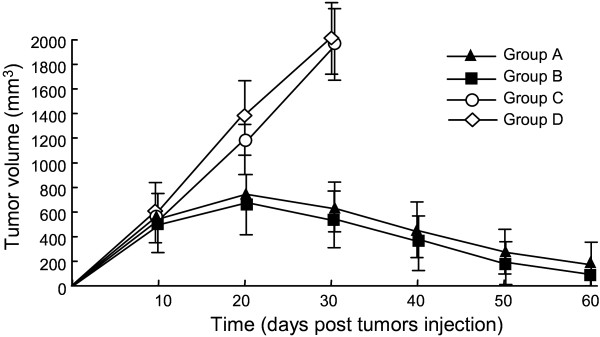
**In vivo antitumor activity of EGFRvIII/CAR**^**+ **^**T cells.** EGFRvIII expressing U87 cells were used for xenograft mouse model. EGFRvIII-bearing BALB/cA-nude mice received different treatments: group A, EGFRvIII/CAR^+^ T cells (IT); group B, EGFRvIII/CAR^+^ T cells (IV); group C, GFP^+^ T cells (IV); group D, PBS (IV). Results are expressed as a mean tumor volume (mm^3^±SD) with *n* = 5 for all groups. The standard deviation (SD) is represented by error bars.

### EGFRvIII/CAR^+^ T cells secreted IFN-γ in an EGFRvIII dependent mechanism

To determine whether EGFRvIII/CAR^+^ T cells become activated and acquire effector cell functions when encountering EGFRvIII target, we performed a cytokine release assay. When co-cultured with EGFRvIII expressing U87 cells, EGFRvIII/CAR^+^ T cells released a substantial amount of IFN-γ, an indicative of T cell activation. In contrast, IFN-γ release remained unchanged in control effector cells (GFP^+^ and non-transduced T cells) or control target cells (parental U87 cells) groups (*P*<0.05, Figure 2B). These results indicate that EGFRvIII/CAR^+^ T cells can be triggered and exert effector cell functions in an EGFRvIII dependent manner, which was consistent with previous findings that the CAR modified T cells expressing a costimulatory signaling domain release increased amount of cytokines [9].

### EGFRvIII/CAR^+^ T cells inhibited the in vivo growth of EGFRvIII expressing glioma cells

We developed a xenograft model by inoculating the EGFRvIII-expressing U87 cells in the flanks of BALB/cA-nude mice. The tumor-loaded mice received injections of 1×10^7^ EGFRvIII/CAR^+^ T cells, GFP^+^ T cells and PBS, respectively. Tumor sizes in mice receiving EGFRvIII/CAR^+^ T cells started to shrink three weeks after adoptive cell transfer, while tumors in other two groups continued to grow. Statistical analysis of the tumor growth curves revealed significant differences between the EGFRvIII/CAR group and control groups (*P*<0.05, Figure 3), confirming the potent antitumor property of the inoculated the EGFRvIII/CAR^+^ T cells in vivo. We excluded the possibility that the antitumor effect of the EGFRvIII/CAR^+^ T cells resulted from their allogeneic effect because the inoculated GFP^+^ T cells did not show any evident effects on tumor growth. In addition, we were convinced by statistical analysis that intravenous injection had a similar efficacy to that of local intratumor injection.

## Discussions

Adoptive transfer of genetically modified T cells shows some advantages over the mobilization of the endogenous T cell repertoire in cancer immunotherapy. The theoretical advantages and technical feasibility of CAR facilitate the development of cancer immunotherapy. The notions that CAR endows T cells antigen specific recognition, activation and proliferation in an MHC independent manner have been consolidated by some pre-clinical studies showing that retargeted T cells can recognize and kill cancer cells expressing tumor associated antigen or specific antigen in vitro and in vivo. Cellular immunotherapy adopting such retargeted T cells has shown significant potential in the treatment of malignant diseases. The mounting data have provided solid support for future clinical application of such therapy in cancers such as leukemia, colorectal colon cancer, and prostate cancer [17-19].

Recent efforts to improve the antitumor efficacy of CAR-based therapy are mainly based on the theory of the two-step T cell activation. Major progress has been made since the introduction of the costimulatory signaling into architecture of CAR. With the in-depth understanding of costimulatory receptors in T cell immune response, several costimulatory molecules were embedded in the CAR and their roles in coordinating antitumor immunity were explored [20]. The observations from other groups and our own have thus far established that the inclusion of costimulatory molecule from B7 receptor family (CD28 or ICOS) results in an increased production of of IFN-γ, TNF-γ, and GM-CSF compared with the CAR with the inclusion of either CD134 or CD137 of TNFR family. CD28 is more potent than other costimulatory molecules with respects to enhanced IL-2 production, improved clonal expansion and persistence of CAR T cells. Finney and colleagues have demonstrated that ICOS in the CAR induces the maximal effect on cell lysis [9]. Thus, we hypothesized that the incorporation of ICOS into CAR favors the antitumor properties of CAR-armed T cells. Our results support our hypotheses: CAR-armed T cells demonstrate efficient killing of tumor cells and abundant Th1 cytokine IFN-γ is released in an EGFRvIII-specific manner. Therefore, our results are consistent with the previous findings and consolidate the notion that the presence of ICOS as an intracellular costimulatory signaling is crucial for enhanced T cell response to tumor cells.

The efficacy of CAR can be affected by many factors including the affinity of the selected scFv, the size of hinge region, the combination of signaling domain(s), the type of modified T cell subsets, etc. The major concern of CAR T cell transfer is the possible recognition of the antigen expressed on normal cells by CAR T cells. Such off-site on-target immune injury causes adverse effects, some of which may be fatal. Therefore, it is important to carefully select the target antigens that are specifically expressed in cancer cells, but not in normal cells. EGFRvIII is a commonly found mutant of EGFR and exclusively expressed in a wide range of cancers. In addition to its tumor specific expression, EGFRvIII is also involved in oncogenic phenotypes and changes the properties of tumorigenicity. Since its discovery, EGFRvIII has become an increasingly attractive molecule for cancer therapy. The EGFRvIII scFv from antibody 3C10 and MR1 was used for CAR construction and the CAR modified T cells demonstrated EGFRvIII-specific tumor cell lysis in vitro and in vivo [21,22]. Recently, Rosenberg’s group analyzed scFv sequences of seven EGFRvIII specific mAbs and assembled the third generation of chimeric antigen receptor (139-28BBZ CAR). The T cells transduced with retroviral EGFRvIII/CAR have been shown an EGFRvIII-specific cell lytic activity [23]. In this study, lentivirus-mediated transduction enriched the EGFRvIII/CAR^+^ T cells to about 70%. Functionality assay demonstrated that these redirected T cells exert efficient cytotoxic T cell response in an EGFRvIII specific manner and secret cytokine IFN-γ in an antigen dependent way. The EGFRvIII/CAR engrafted T cells pave a way for antitumor in animal model as well as in clinical settings.

In our study, we used CD3^+^ T cells, instead of purified CD8^+^ CTLs only, to investigate the performance of CAR because CD4^+^ T cells have been shown to augment the function of CD8^+^ T cells. The results of our EGFRvIII-bearing mouse model demonstrates that CD3^+^ T cells transduced with EGFRvIII/CAR have significantly higher antitumor activity than the T cells in control groups, which supports the theory that adoptive transfer of mixed populations of antigen-specific CD8^+^ and CD4^+^ T cells promotes overall antitumor immunity. With regards to the administration route of T cells, both intratumor injection and venous injection show similar efficacy. For clinical purpose, engraftment of adoptively transferred T cells in host is a major challenge for achieving therapeutic benefit. Ongoing studies are exploring optimal combinations of costimulatory molecules and T cell subsets with long-term cytotoxicity [24]. Also, most of mice in groups of the EGFRvIII/CAR^+^ T cells and GFP^+^ T cells suffered from graft versus host diseases (GVHD) to some extent. The symptoms of GVHD usually started 5 weeks after T cells infusion, including less activity, ruffled fur, skin rash, hunched back and weight loss. The lethality due to the transfusion-associated GVHD was rare and there was no difference between mice treated with EGFRvIII/CAR^+^ T cells and GFP^+^ T cells with regard to the severity of GVHD.

## Conclusion

Our study demonstrates that the EGFRvIII/CAR-modified T cells are capable of destroying glioma cells efficiently in an EGFRvIII specific manner and release IFN-γ in an antigen dependent manner. The specific recognition and effective killing activity of the EGFRvIII-directed T cells with ICOS signaling domain provides a basis for further studies in clinical application of cancer treatment.

## Abbreviations

CAR: Chimeric antigen receptor; ICOS: Inducible costimulator; EGFRvIII: Epidermal growth factor variant III; AIT: Adoptive immunotherapy; scFv: single chain variable fragment; TCR: T cell receptor; CTLs: Cytotoxic T lymphocytes; GFP: Green fluorescent protein; MHC: Major histocompatibility complex; TNFR: Tumor necrosis factor receptor; GVHD: Graft versus host diseases

## Competing interests

The authors declare no competing financial interests in relation to this work.

## Authors’ contributions

SCJ and YYX carried out most of the molecular and cellular experiments and drafted the manuscript. HEQ and CN performed the flow cytometry and Western blot analysis. WYF, WY and ZYY did the in vivo experiments in animal model. ZLM carried out the statistical analysis. CJ, GP and WAJ finished experiments related to EGFRvIII scFv and participated in discussion of the research. HSY designed the research and wrote the manuscript. All authors read and approved the manuscript.
